# Study of the Association of Semen Parameters With Embryo Quality in a Tertiary Care Center in Bihar: A Retrospective Study

**DOI:** 10.7759/cureus.68209

**Published:** 2024-08-30

**Authors:** Sangeeta Kumari, Kalpana Singh, Abhigyan Kumar, Shubhanti Kumari, Aditi Raina

**Affiliations:** 1 Reproductive Medicine, Indira Gandhi Institute of Medical Sciences (IGIMS), Patna, IND; 2 General Medicine, Netaji Subhas Medical College and Hospital, Patna, IND

**Keywords:** assisted reproductive technology, sperm morphology, sperm motility, sperm count, embryo quality, semen quality

## Abstract

Introduction

Semen quality, characterized by parameters such as sperm count, motility, and morphology, determines successful fertilization and subsequent embryo health. This study investigates the impact of sperm parameters on embryo quality in assisted reproductive technology (ART).

Methods

The study utilized a retrospective design with 194 male and 194 female participants who underwent fertility treatment from October 2020 to May 2024. Semen analysis included assessment of sperm count, motility, and morphology. Embryo quality was evaluated on days three and five post-fertilizations based on morphological criteria. Statistical analyses, including one-way ANOVA and chi-square tests, explored relationships between semen parameters and embryo quality.

Results

The study included 388 participants (males: 194 and females: 194). Female participants had a mean age of 31.0 ± 4.6 years and a mean BMI of 23.1 ± 5.3 kg/m², while males had a mean age of 36.6 ± 5.4 years and a mean BMI of 22.7 ± 2.8 kg/m². Paternal age and BMI showed no significant association (p > 0.05) with embryo quality. However, sperm quality parameters such as sperm count, motility, and morphology demonstrated significant associations (p < 0.05) with embryo quality on both day three and day five, indicating that abnormal sperm parameters were linked to poorer embryo quality. Factors such as alcohol consumption, smoking, tobacco use, living in industrial areas, and tea/coffee consumption showed no significant association with embryo quality.

Conclusion

The findings of the study emphasize the importance of comprehensive semen analysis in fertility assessments and highlight opportunities for improving ART outcomes through targeted interventions and further mechanistic research.

## Introduction

The quality of semen influences not only the success rate of fertilization but also the subsequent development of the embryo [1]. Semen quality is assessed based on various parameters, including sperm count, motility, morphology, and DNA integrity [2]. Each of these parameters helps in determining the fertilizing potential of the sperm and the health of the resulting embryo. Higher sperm concentrations increase the probability of successful fertilization by providing more opportunities for sperm to reach and penetrate the egg. This is especially critical in natural conception and in intrauterine insemination (IUI) procedures, where sperm must travel through the female reproductive tract to reach the egg. Sperm motility is another factor in semen quality, which describes the ability of sperm to move actively and efficiently [3]. Motility is categorized into progressive motility, where sperm move forward in a straight line or large circles, and non-progressive motility, where movement is slow or circular [4]. Progressive motility is essential for sperm to navigate through the cervical mucus, uterine cavity, and fallopian tubes to reach the egg. High motility is correlated with increased chances of natural conception and successful ART outcomes [5].

Sperm morphology refers to the shape and structural features of sperm [6]. Normal morphology is characterized by an oval head, an intact midpiece, and a long tail. Abnormalities in any of these structures can impair the sperm’s ability to fertilize an egg [7]. Assessing the percentage of sperm with normal morphology helps predict fertilization potential. The initial interaction between sperm and egg is crucial for successful fertilization. High-quality sperm are better equipped to penetrate the protective layers of the egg and initiate the fertilization process. Following fertilization, the quality of sperm impacts the development of the embryo. Embryos derived from sperm of high quality are more likely to develop normally, leading to successful implantation and healthy pregnancy outcomes [8].

Advancements in fertility treatments, such as in vitro fertilization (IVF) and intracytoplasmic sperm injection (ICSI), have been driven by an improved understanding of semen quality [9]. In IVF, selecting high-quality sperm enhances the likelihood of successful fertilization and embryo development. ICSI, which involves the direct injection of a single sperm into an egg, is particularly beneficial for severe male infertility cases, ensuring that even sperm with suboptimal motility or morphology can achieve fertilization. Despite such advances, the underlying mechanisms by which semen quality affects embryogenesis remain inadequately understood. This study aims to elucidate the relationship between various parameters of semen quality and their impact on embryo quality.

## Materials and methods

Study design and recruitment

This study employed a retrospective design to investigate the relationship between semen quality parameters and embryo quality. It aimed to assess how variations in semen parameters (sperm count, motility, morphology) influence the quality of embryos produced through ART. Women in the age group of 21 to 40 years undergoing IVF treatment during the period from October 2020 to May 2024 in the Department of Reproductive Medicine, Indira Gandhi Institute of Medical Sciences (IGIMS), Patna, Bihar, and their partners were recruited in the study. All the participants provided their signed informed consent. Patients not falling into the specified age category and those with incomplete data were excluded from the study. The study included a total of 388 participants, comprising 194 females and 194 males.

Semen analysis

Semen samples were collected from male participants following a period of abstinence recommended by the clinic (typically two to five days). Semen analysis was performed according to World Health Organization (WHO) guidelines published in 2021. Parameters assessed included sperm count: the number of spermatozoa per milliliter of semen; sperm motility: categorized into progressive motility (sperm moving forward in a straight line or large circles) and non-progressive motility (slow or circular movement); sperm morphology: evaluation of sperm shape and structure, focusing on the head, midpiece, and tail.

Assessment of embryo quality

Quality assessment and grading of the embryos was based on the Gardner grading guide [10]. Day three embryos were assessed for quality on the basis of morphological criteria (blastomere number, blastomere regularity, nuclear content, and fragmentation rate). The following grading system was used: good (equally sized symmetrical blastomeres without fragmentation), moderate (unevenly sized blastomeres without fragmentation), and poor (embryos with 50% or more fragmentation). Day five embryos were assessed for quality on the basis of morphological criteria such as inner cell mass formation, trophectoderm layer formation, blastocoel cavity formation, and fragmentation. The following grading system used followed: Grade A (good): numerous and tightly packed inner cell mass; trophectoderm is many-celled and organized in the epithelium. Grade B (moderate): several and loosely packed inner cell mass; trophectoderm is several-celled and organized in the loose epithelium. Grade C (poor): few and unorganized inner cell mass and trophectoderm

Ethical considerations

Ethical approval was obtained from the Institutional Ethics Committee Indira Gandhi Institute of Medical Sciences (IGIMS), Patna, Bihar, India before initiating the study (1321/IEC/IGIMS/2023).

Statistical analysis

Statistical analysis was performed using SPSS software Version 21 (IBM Corp., Armonk, NY). Shapiro-Wilk test was employed for checking the normality of the data. Continuous variables were represented as mean ± SD and categorical variables were expressed as frequency (%). Chi-square tests were employed to assess associations between categorical variables and embryo quality. One-way ANOVA was used to compare paternal age between different embryo quality groups. P-values less than 0.05 were considered to be significant.

## Results

The study included a total of 388 participants, comprising 194 females and 194 males. Female participants had a mean age of 31.0 ± 4.6 years. Their mean BMI was 23.1 ± 5.3 kg/m^2^. Male participants had a mean age of 36.6 ± 5.4 years with a mean BMI of 22.7 ± 2.8 kg/m^2^ (Table 1).

**Table 1:** Demographic characteristics of the participants

	Demographic characteristic	Mean	SD
Female (n = 194)	Age (years)	31.0	4.6
	Weight (kg)	57.3	9.0
	Height (cm)	157.0	7.7
	BMI	23.1	5.3
Male (n = 194)	Age (years)	36.6	5.4
	Weight (kg)	66.9	4.6
	Height (cm)	157.1	8.0
	BMI	22.7	2.8

Among the male partners, a substantial proportion reported smoking and alcohol consumption (74 participants each), while fewer reported tobacco use (29 participants). Living in industrial areas was reported by eight participants, and only two participants had ever lived in high-altitude areas. Consumption of tea or coffee was reported by 64 participants (Table 2).

**Table 2:** Male lifestyle and environmental factors

Male parameters	Yes (%)	No (%)
Smoking	74 (38.14)	120 (61.86)
Alcohol	74 (38.14)	120 (61.86)
Tobacco	29 (14.95)	165 (85.05)
Living in industrial area	8 (4.12)	186 (95.88)
Ever lived in high altitude	2 (1.03)	192 (98.97)
Tea/coffee	64 (32.99)	130 (67.01)

Among the females, primary subfertility was observed in 52 participants, indicating difficulties in achieving a first pregnancy within the specified timeframe despite regular unprotected intercourse. Primary infertility, characterized by the inability to conceive after at least one year of unprotected intercourse, was noted in 59 participants. Secondary subfertility, where participants had previous pregnancies but encountered challenges conceiving again, was identified in 34 individuals. Similarly, secondary infertility, involving difficulties in achieving subsequent pregnancies after prior successful pregnancies, was reported in 31 participants. A small number of participants, five in total, were classified as nulliparous, indicating they had never given birth. Additionally, 13 participants had an unknown history regarding their fertility status.

Effect of paternal age and BMI

No significant association was observed between paternal age (good quality embryo group: 32 ± 4.4 years, moderate quality embryo group: 34 ± 2.8 years, and poor quality embryo group: 33 ± 3.9 years; p=0.08) and BMI with embryo quality (Table 3 and Table 4).

**Table 3:** Association of male factors with day three embryo quality A chi-square test was employed to assess associations between categorical variables and embryo quality. P-values less than 0.05 were considered to be significant.

	Good (n = 70)	Moderate (n = 73)	Poor (n = 51)	P-value
BMI (kg/m^2^)				
Underweight (<18.5)	14	11	7	0.34
Normal (18.5 - 24.9)	49	52	28	
Overweight/obese (≥25)	7	10	16	
Sperm count (millions/milliliter)				
Abnormal <15	22	28	36	<0.001
Normal ≥15	48	45	15	
Sperm motility (%)				
Abnormal <40	18	18	42	<0.001
Normal ≥40	52	55	9	
Sperm morphology (%)				
Abnormal <4	13	50	46	0.03
Normal ≥4	57	23	5	
Alcohol consumption (n)				
Yes	34	25	33	0.38
No	36	48	18	
Smoking (n)				
Yes	34	22	35	0.47
No	36	51	16	
Tobacco (n)				
Yes	8	8	18	0.33
No	62	65	33	
Living in an industrial area (n)				
Yes	3	4	3	0.45
No	67	69	48	
Consumption of tea/coffee (n)				
Yes	22	19	33	0.56
No	48	54	18	

**Table 4:** Association of male factors with day five embryo quality A chi-square test was employed to assess associations between categorical variables and embryo quality. P-values less than 0.05 were considered to be significant.

	Good (n = 63)	Moderate (n = 33)	Poor (n = 98)	P-value
BMI (kg/m^2^)				
Underweight (<18.5)	4	4	8	0.33
Normal (18.5 - 24.9)	55	23	75	
Overweight/obese (≥25)	4	6	15	
Sperm Count (millions/milliliter)				
Abnormal <15	0	2	12	<0.001
Normal ≥15	63	31	86	
Sperm motility (%)				
Abnormal <40	15	13	38	<0.001
Normal ≥40	48	20	60	
Sperm morphology (%)				
Abnormal <4	18	22	91	0.02
Normal ≥4	45	11	7	
Alcohol consumption (n)				
Yes	27	11	36	0.34
No	36	22	62	
Smoking (n)				
Yes	28	12	34	0.55
No	35	21	64	
Tobacco (n)				
Yes	11	28	18	0.44
No	52	5	80	
Living in an industrial area (n)				
Yes	4	5	4	0.37
No	59	28	94	
Consumption of tea/coffee (n)				
Yes	24	2	38	0.67
No	39	31	60	

Effect of sperm count

Assessment of sperm quality parameters including sperm count, motility, and morphology was done. Fourteen out of 194 participants (7.2%) had a sperm count below 15 million per milliliter, falling into the abnormal category. The majority of participants (92.8%) had normal sperm counts (≥15 million per milliliter). A significant (p < 0.001) association of sperm count was observed with both day three and day five embryo quality (Table 3 and Table 4). Participants with abnormal sperm count had poor-quality embryos compared to participants with normal sperm count.

Effect of sperm motility

Regarding sperm motility, 66 participants reported abnormal values with an average of 25.03 ± 10.62% motile sperm only. Whereas, 128 participants reported normal values with an average of 56.60 ± 13.64% motility. A significant (p < 0.001) effect of sperm motility on day three and day five embryo quality was observed (Table 3 and Table 4). Participants with abnormal sperm motility had poor quality embryos compared to participants with normal sperm motility.

Effect of sperm morphology

In terms of sperm morphology, participants exhibited an average of 1.0 abnormal forms per ejaculation. The Majority of participants (131 out of 194) displayed abnormal morphology. Both day three (p = 0.03) and day five (p = 0.02) embryo quality was significantly associated with sperm morphology (Table 3 and Table 4). Participants with abnormal sperm morphology had poor quality embryos compared to participants with normal sperm morphology.

Factors such as alcohol consumption, smoking, tobacco use, living in industrial areas, and consumption of tea/coffee did not have any significant association with embryo quality (Table 3 and Table 4).

## Discussion

In the present study, we observed a significant association between sperm count, motility, morphology, and embryo quality at both day three and day five post-fertilization. Participants with lower sperm counts (<15 million per milliliter) consistently exhibited poorer embryo quality compared to those with normal sperm counts (≥15 million per milliliter). This finding emphasizes the crucial role of sperm concentration in successful embryo development. A higher sperm count likely provides more spermatozoa available for fertilization, increasing the chances of successful embryo formation and subsequent development. Furthermore, our findings revealed a strong correlation between sperm motility and embryo quality. Participants with higher percentages of motile sperm showed significantly better embryo quality on both assessed days. Progressive motility, in particular, plays a crucial role in facilitating sperm movement through the female reproductive tract to reach the egg. This result supports the notion that motility is a key predictor of fertilization success in ART. The ability of sperm to actively navigate toward the egg is paramount for successful embryo development post-fertilization. One study that investigated male factors like sperm quality and paternal age in conventional IVF and ICSI outcomes showed that severe oligospermia negatively impacted fertilization and blastulation rates in ICSI. Reduced motility affected IVF outcomes, correlating with lower fertilization and embryo counts. Additionally, advanced paternal age, especially above 51 years, decreased pregnancy rates in both IVF and ICSI, particularly when coupled with maternal age over 37 [11]. Contrastingly, another study showed that advanced paternal age showed a minor impact on fertilization rates but did not affect other outcomes like top-quality blastocyst formation or ongoing pregnancy rates. However, this study also reported that poor sperm motility (<5%) correlated negatively with fertilization rates [12]. Similar to these findings, we observed a significant positive association of normal sperm motility with good embryo quality.

The impact of sperm morphology on embryo quality varied depending on the developmental stage assessed. Maternal factors have long been acknowledged as influential in embryo quality, while the contribution of male factors, particularly sperm morphology, is still being explored [13]. Sperm showing abnormalities in the post-acrosomal region or containing cytoplasmic droplets tend to produce lower-quality embryos [14]. Coban et al. observed a link between sperm morphology and embryo aneuploidy, using donor oocytes to isolate paternal influences. They categorized 1165 embryos based on Kruger's strict criteria for sperm morphology, noting that higher scores correlated with improved fertilization rates, although not statistically significant. Research also indicates that sperm morphology deteriorates with increasing paternal age, with older men showing higher rates of abnormal sperm. Despite this, Coban et al. did not find a clear association between advanced paternal age and total aneuploidy rates or fertilization success in their studies [15].

In the present study, it was observed that abnormal sperm morphology significantly affected day three and day five embryo quality. This suggests that abnormalities in sperm structure, such as head shape or tail defects, may compromise the embryo's developmental potential later in its progression. Morphologically normal sperm are better equipped to penetrate the egg and support early embryonic growth and cell division, whereas abnormalities may hinder these critical processes as development progresses. Previous research indicates a negative correlation between sperm morphology and oocyte fertilization and suggests lower pregnancy rates with decreasing sperm morphology. However, recent studies have shown successful pregnancies occurring even in cases of highly abnormal sperm morphology or none at all. This has led to uncertainty among clinicians, as some doubt the predictive value of specific morphology thresholds, such as the <4% normal morphology criterion [16]. In IVF, some studies support the predictive value of morphology, while others demonstrate no significant impact on pregnancy outcomes, even in cases of severe teratozoospermia. This inconsistency challenges the notion that morphology alone should dictate treatment decisions like ICSI, where sperm are directly injected into eggs [16]. Studies comparing IVF and ICSI outcomes in men with severe teratozoospermia have shown mixed results, with some indicating no significant difference in fertilization or pregnancy rates between the two techniques [17]. These studies suggest that while sperm morphology may influence IVF outcomes under certain conditions, such as very low percentages of normal forms (< 4%), its impact is not consistently decisive across all cases. Factors like motility and overall sperm count post-wash may prove more significant in predicting success in IUI, whereas in IVF and ICSI, morphology alone may not be the sole determinant.

Limitations

This study follows a retrospective design, which restricted the ability to establish causality between semen parameters and embryo quality. Additionally, the small sample size and demographic characteristics of participants may limit the generalizability of findings to broader populations.

## Conclusions

Participants with higher sperm counts, better motility, and morphology exhibited superior embryo quality, highlighting the importance of these parameters in successful fertilization and early embryo development. These findings emphasize the need for comprehensive semen analysis in fertility assessments. Future research should explore the underlying mechanisms linking semen parameters to embryo development. Understanding these mechanisms could lead to targeted interventions to improve semen quality and enhance ART success rates.
